# Hyposmia as a marker of (non-)motor disease severity in Parkinson’s disease

**DOI:** 10.1007/s00702-019-02074-0

**Published:** 2019-09-12

**Authors:** Dareia S. Roos, Jos W. R. Twisk, Pieter G. H. M. Raijmakers, Richard L. Doty, Henk W. Berendse

**Affiliations:** 1 Department of Neurology, Amsterdam UMC, Vrije Universiteit Amsterdam, De Boelelaan 1117, Amsterdam, The Netherlands; 2 Department of Epidemiology and Biostatistics, Amsterdam UMC, Vrije Universiteit Amsterdam, De Boelelaan 1117, Amsterdam, The Netherlands; 3 Department of Radiology and Nuclear Medicine, Amsterdam UMC, Vrije Universiteit Amsterdam, De Boelelaan 1117, Amsterdam, The Netherlands; 4 Smell and Taste Center, Perelman School of Medicine, University of Pennsylvania, Philadelphia, USA

**Keywords:** Parkinson’s disease, Olfaction, Disease severity, Motor symptoms, Non-motor symptoms

## Abstract

The aim of this study was to evaluate the relationship of hyposmia in Parkinson’s disease (PD) with other motor and non-motor symptoms and with the degree of nigrostriatal dopaminergic cell loss. A total of 295 patients with a diagnosis of PD were included. Olfactory function was measured using the University of Pennsylvania Smell Identification Test (UPSIT). Motor symptoms were rated using the Unified Parkinson’s Disease Rating Scale motor subscale (UPDRS III). To evaluate other non-motor symptoms, we used the Mini-Mental State Examination (MMSE) as a measure of global cognitive function and validated questionnaires to assess sleep disturbances, psychiatric symptoms, and autonomic dysfunction. A linear regression model was used to calculate correlation coefficients between UPSIT score and motor and non-motor variables [for psychiatric symptoms a Poisson regression was performed]. In a subgroup of patients (*n* = 155) with a dopamine transporter (DaT) SPECT scan, a similar statistical analysis was performed, now including striatal DaT binding. In the regression models with correction for age, sex, disease duration, and multiple testing, all motor and non-motor symptoms were associated with UPSIT scores. In the subgroup of patients with a DaT-SPECT scan, there was a strong association between olfactory test scores and DaT binding in both putamen and caudate nucleus. Hyposmia in PD is associated with various motor and non-motor symptoms, like cognition, depression, anxiety, autonomic dysfunction and sleep disturbances, and with the degree of nigrostriatal dopaminergic cell loss. This finding adds further confirmation that hyposmia holds significant promise as a marker of disease progression.

## Introduction

Hyposmia is one of the most common non-motor symptoms in Parkinson’s disease (PD), with a reported prevalence of up to 90% (Doty et al. 1988; Haehner et al. 2009; Hawkes et al. 1997), that may precede the first motor symptoms by several years (Ponsen et al. 2004; Ross et al. 2008). Given the increasing load of Lewy body pathology in the olfactory system with advancing neuropathological stages of PD (Braak et al. 2003; Del Tredici et al. 2002), one would expect a progressive decline in olfactory function that correlates with other clinical markers of disease progression. However, previous clinical studies have yielded conflicting data. Although some studies showed a correlation between disease duration and olfactory function (Deeb et al. 2010; Ramjit et al. 2010), most studies have failed to find this correlation (Cavaco et al. 2015; Haehner et al. 2009; Hawkes et al. 1997; Herting et al. 2008; Lee et al. 2015; Masala et al. 2018). In the only longitudinal study, olfactory loss in PD remained stable over time (Doty et al. 1988). Similarly, while in some studies hyposmia was associated with disease severity as measured with the Unified PD Rating Scale motor subscale (UPDRS III) (Cavaco et al. 2015; Deeb et al. 2010; Lee et al. 2015; Masala et al. 2018; Tissingh et al. 2001), others found no association between the degree of hyposmia and disease stage or severity (Boesveldt et al. 2008; Doty et al. 1988; Haehner et al. 2009; Herting et al. 2008; Ramjit et al. 2010; Siderowf et al. 2005).

So far, only few studies have focused on the relationship between olfactory function and non-motor symptoms in PD. Correlations have been reported between hyposmia and apathy (Cramer et al. 2010; Hong et al. 2015), cognitive dysfunction (Domellof et al. 2017; Fullard et al. 2016), and autonomic failure (Chen et al. 2015; Fullard et al. 2016; Goldstein et al. 2010). In a few studies in which striatal dopamine transporter (DaT) binding was used as a marker of the nigrostriatal dopaminergic deficit, hyposmia was associated with lower striatal DaT binding (Bohnen et al. 2007; Siderowf et al. 2005).

The aim of this study was to examine the association between olfactory function and various motor and non-motor symptoms and striatal DaT binding in a large cohort of PD patients.

## Methods

### Study population

For this study, we used the clinical and dopamine transporter imaging data of 295 PD patients collected as part of their routine clinical care during visits to the outpatient clinic for movement disorders at the VU University Medical Center (VUmc). All patients were seen between May 2008 and February 2014 by a neurologist specialized in movement disorders and fulfilled the clinical diagnostic criteria of the UK PD Society Brain Bank. Olfactory testing using the University of Pennsylvania Smell Identification Test (UPSIT) has been routinely performed in all-new referred patients seen at the outpatient clinic. We selected those with a complete UPSIT and a diagnosis of PD. The mean age of our patients was 65.3 years, with a mean disease duration of 5.3 years (Table 1).

**Table 1:** Characteristics of the study population

All participants	PD patients (*n* = 295)	PD patients with DaT-SPECT (*n* = 155)
Mean age, years	65.3 (10.4)	65.4 (10.7)
Males *n* (%)	179 (60.7%)	95 (61.3%)
Mean disease duration, years	5.3 (4.9)	3.7 (3.7)
Modified H & Y stage, *n* (%)		
1	31 (10.5%)	19 (12.3%)
1.5	21 (7.1%)	13 (8.4%)
2	118 (40.0%)	69 (44.5%)
2.5	65 (22.0%)	32 (20.6%)
3	35 (11.9%)	16 (10.3%)
4	15 (5.1%)	3 (1.9%)
5	10 (3.4%)	3 (1.9%)
UPSIT	20.3 ± 7.3	21.2 ± 7.2
UPDRS III	27.3 ± 13.6 (*n* = 288)	25.2 ± 12.4 (*n* = 153)
MMSE	27.3 ± 3.3 (*n* = 280)	27.6 ± 3.3 (*n* = 148)
SCOPA-SLEEP	23.4 ± 7.8 (*n* = 286)	22.3 ± 6.7 (*n* = 150)
SCOPA-PC	1.1 ± 1.5 (*n* = 272)	0.9 ± 1.2 (*n* = 147)
SCOPA-AUT	35.6 ± 8.7 (*n* = 281)	34.6 ± 8.5 (*n* = 150)
BDI	11.6 ± 8.2 (*n* = 285)	10.5 ± 7.6 (*n* = 151)
BAI	15.0 ± 10.0 (*n* = 280)	12.7 ± 8.7 (*n* = 146)

Data are shown as numbers (%) or mean (SD)

*PD* Parkinson’s disease, *H&Y* Hoehn & Yahr, *DaT-SPECT* dopamine transporter single-photon emission computed tomography

Disease duration was measured from the onset of subjective motor symptoms. When a patient had experienced motor symptoms for a period between 6 and 12 months, disease duration was defined as 1 year; in case of a duration of subjective motor symptoms shorter than 6 months, disease duration was defined as zero years. Disease stage was defined using the modified Hoehn & Yahr staging [H&Y (Goetz et al. 2004)]. The majority of patients were in stages 2 and 2.5. Most patients used levodopa monotherapy, a dopamine agonist or a combination of levodopa and a dopamine agonist. In a subgroup of 155 patients, a DaT Single-Photon Emission Computed Tomography (DaT-SPECT) scan was available. The mean age of this subgroup was 65.4 years, with a mean disease duration of 3.7 years (Table 1). All patients had provided written informed consent to store the clinical and imaging data obtained as part of their routine clinical care in a database to be used later for scientific research.

### Clinical profiling

The Unified Parkinson’s Disease Rating Scale motor subscale (UPDRS III; range 0–108) (Fahn and Elton 1987) was used as a measure of motor symptom severity. Motor symptom severity was rated while patients were on medication. Cognitive function was tested using the Mini-Mental State Examination (MMSE) (Folstein et al. 1975). Sleep disturbances were assessed using the SCales for Outcomes in PArkinson’s disease for sleep (SCOPA-SLEEP) (Marinus et al. 2003). To evaluate psychiatric symptoms, the SCales for Outcomes in PArkinson’s disease Psychiatric Complications (SCOPA-PC) (Visser et al. 2007) was used. Autonomic failure was assessed by means of the Scales for Outcomes in PArkinson’s disease Autonomic dysfunction (SCOPA-AUT) (Visser et al. 2004). The questions in the SCOPA-AUT aimed at sexual functioning were excluded from our analysis, as too many data were missing. Depressive symptoms and anxiety symptoms were screened with the Beck Depression Inventory (BDI) (Beck et al. 1961) and the Beck Anxiety Inventory (BAI) (Beck et al. 1988), respectively. For all tests except the MMSE, a higher score means more severe symptoms. Tests were either self-administered (SCOPA-SLEEP, SCOPA-AUT, BDI and BAI), or administered by a trained physician or nurse specialist (UPDRS III, modified H&Y, MMSE, SCOPA-PC) in the same period as the UPSIT was performed.

### Olfactory testing

The Dutch version of the UPSIT was used as a measure of olfactory function (Doty et al. 1984). The UPSIT is a self-administered forced-choice odour identification test consisting of 40 odorized items. After scratching a patch containing microcapsules filled with odorant, the subject sniffs the released odorant and chooses the best alternative from four response alternatives. The test was self-administered at home. Completeness was checked in the hospital, and when necessary the patient was asked to complete any missing items. The total score was calculated by adding up all correct answers.

### Nuclear imaging: DaT-SPECT

[^123^I]*N*-ω-fluoropropyl-2β-carbomethoxy-3β-(4-idodophenyl)nortropane ([^123^I]FP-CIT) SPECT was used to measure dopamine transporter (DaT) binding. To block uptake of free radioactive iodide in the thyroid, all patients received potassium perchlorate orally. [^123^I]FP-CIT was injected intravenously at an approximate dose of 185 MBq (specific activity > 185 MBq/nmol; radiochemical purity > 99%). Three hours after injection, the images were acquired. Slices were made over 300-s periods, during which the head of the patient was positioned in the camera. Semi-quantitative analysis using a standard template with regions of interest (ROI) was used for analysing the SPECT images. Tracer binding values in the individual regions were used to calculate a tracer binding ratio of DaT binding in the putamen or caudate nucleus and the occipital cortex, which was used as a reference area. The least and most affected side of the putamen and the caudate nucleus were used in the statistical analysis.

### Statistical analysis

To verify whether the percentage of patients with an olfactory deficit was comparable to that known from the literature, we calculated this percentage using previously established optimal discrimination criteria for the UPSIT (Doty et al. 1995). These criteria are adjusted for age and gender. To analyse the relationship between olfactory function and each of the motor and non-motor variables, a linear regression analysis was used. For each variable, three analyses were performed: the first crude, the second adjusted for age and gender, and the third further adjusted for disease duration. Data from the SCOPA-SLEEP and BDI were first log transformed, because of non-normally distributed residuals. To analyse the SCOPA-PC a Poisson regression was performed. In the group of patients with an available DaT-SPECT scan, the same three analyses were performed with UPSIT, UPDRS III and MMSE as dependent variables. The data were analyzed with SPSS 22.0 (SPSS Inc., Chicago, IL, USA). The significance level was set at 0.05 and to adjust for multiple testing a Bonferroni correction was applied.

## Results

### Correlations with clinical measures of motor and non-motor function

**Fig. 1:**
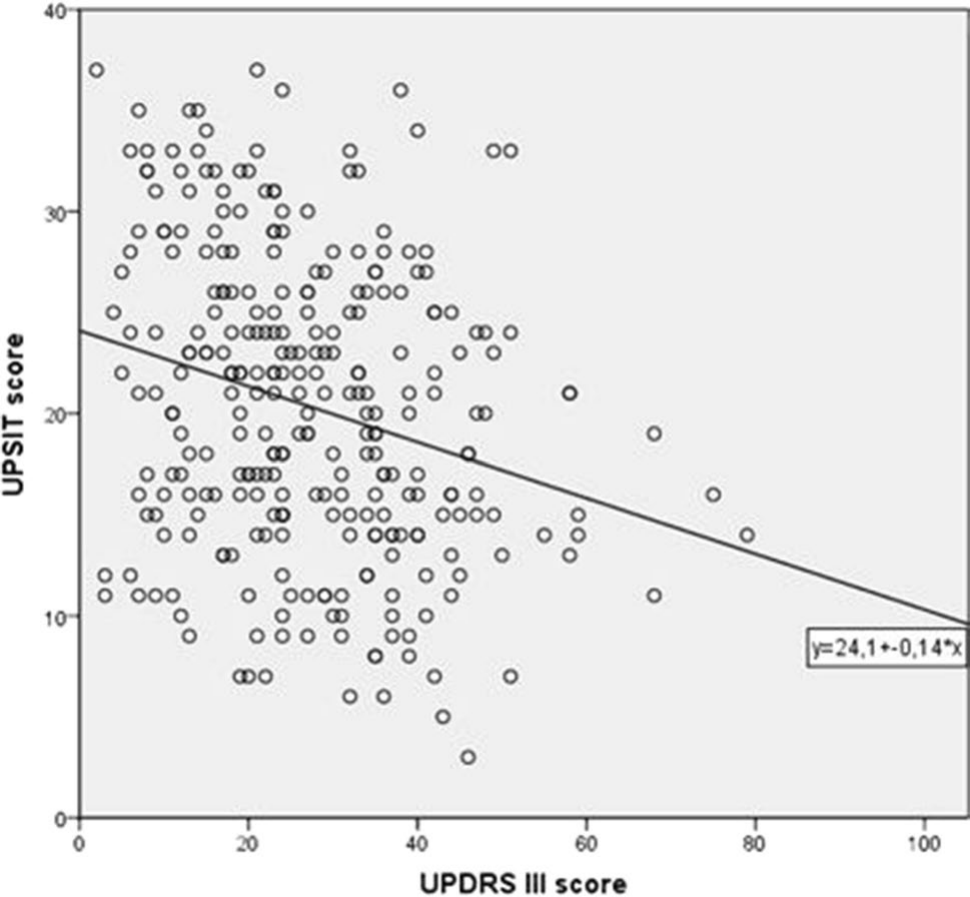
Scatter plot illustrating the correlation between UPSIT scores and UPDRS III motor scores

**Fig. 2:**
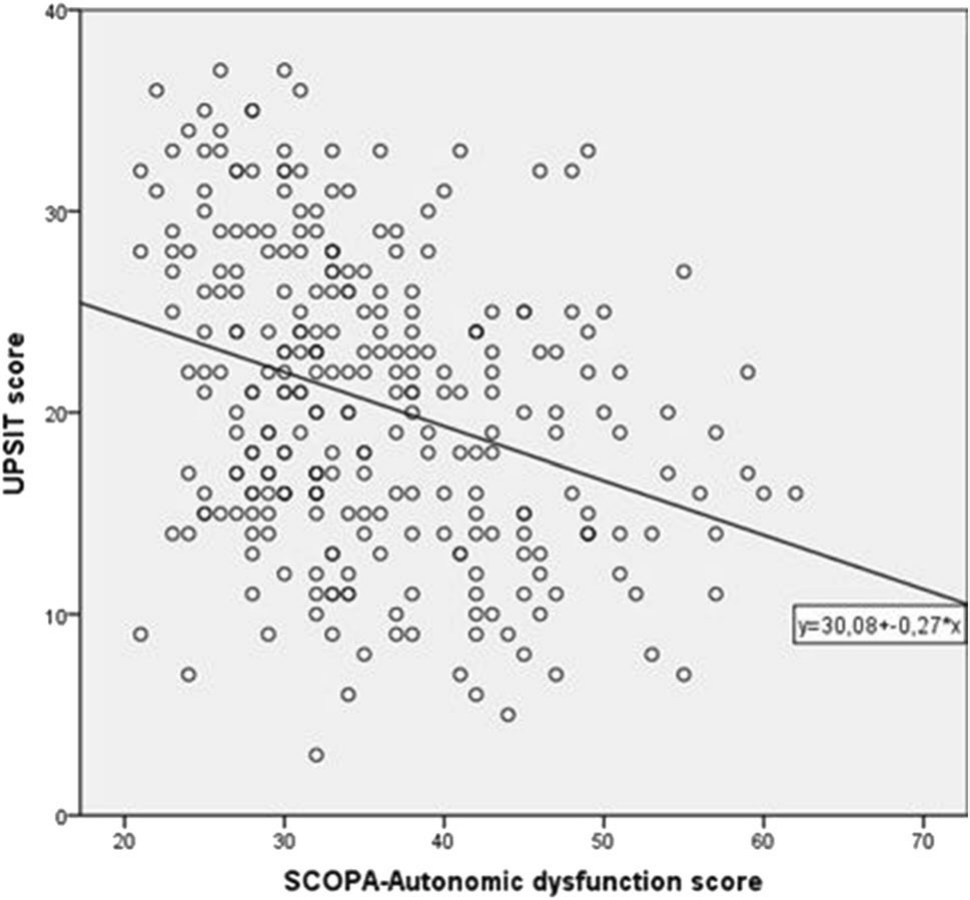
Scatter plot illustrating the correlation between UPSIT scores and SCOPA-Autonomic dysfunction scores

In our total group of 295 PD patients, 82.7% were hyposmic or anosmic when compared to the optimal discrimination criteria established by Doty et al. (1995). Motor function as measured with UPDRS III was significantly associated with olfaction, also in the models in which we adjusted for sex and age (Table 2; Fig. 1). For each of the non-motor symptoms we assessed, test scores were significantly associated with UPSIT scores. The strongest crude association of olfactory function was with SCOPA-AUT scores (Fig. 2). As expected, adjustment for age and sex attenuated all observed associations, although all associations remained statistically significant, except for the correlation with MMSE. Further adjustment for disease duration did not influence the regression coefficients, besides correlation with MMSE and UPDRS III, which indicates that the influence of disease duration is negligible.

**Table 2:** Results of the linear regression analyses relating UPSIT score to various motor and non-motor variables

		*B*	B (95% CI)	*p*
UPDRS III	Model 1	− 0.254	− 0.466 (− 0.673; − 0.259)	< 0.001*
	Model 2	− 0.157	− 0.289 (− 0.499; − 0.078)	0.007*
	Model 3	− 0.129	− 0.237 (− 0.442; − 0.032)	0.023
MMSE	Model 1	0.208	0.093 (0.041; 0.145)	< 0.001*
	Model 2	0.143	0.064 (0.010; 0.118)	0.020
	Model 3	0.122	0.055 (0.001; 0.109)	0.045
SCOPA-SLEEP^a^	Model 1	− 0.185	− 0.003 (− 0.006; − 0.001)	0.002*
	Model 2	− 0.197	− 0.004 (− 0.006; − 0.001)	0.002*
	Model 3	− 0.163	− 0.003 (− 0.005; − 0.001)	0.007*
SCOPA-PC	Model 1		0.949^b^ (0.934; 0.965)	< 0.001*
	Model 2		0.950^b^ (0.934; 0.966)	< 0.001*
	Model 3		0.953^b^ (0.937; 0.969)	< 0.001*
SCOPA-AUT	Model 1	− 0.318	− 0.376 (− 0.508; − 0.244)	< 0.001*
	Model 2	− 0.249	− 0.294 (− 0.431; − 0.157)	< 0.001*
	Model 3	− 0.204	− 0.241 (− 0.366; − 0.116)	< 0.001*
BDI^1^	Model 1	− 0.203	− 0.010 (− 0.015; − 0.004)	0.001*
	Model 2	− 0.190	− 0.009 (− 0.015; − 0.003)	0.002*
	Model 3	− 0.168	− 0.008 (− 0.014; − 0.002)	0.006*
BAI	Model 1	− 0.203	− 0.276 (− 0.434; − 0.119)	0.001*
	Model 2	− 0.208	− 0.283 (− 0.447; − 0.119)	0.001*
	Model 3	− 0.175	− 0.238 (− 0.395; − 0.081)	0.003*

*B *= standardized coefficient. B = unstandardized coefficient. Model 1 for UPSIT only. Model 2: adjusted for sex and age. Model 3: as model 2 with adjustment for disease duration

*UPSIT* University of Pennsylvania Smell Identification Test, *UPDRS III* Unified Parkinson’s Disease Rating Scale motor subscale, *MMSE* Mini-Mental State Examination, *SCOPA* SCales for Outcomes in PArkinson’s Disease, *PC* psychiatric complications, *AUT* autonomic dysfunction, *BDI* Beck Depression Inventory, *BAI* Beck Anxiety Inventory

^a^Log transformed data

^b^Exp(B) with Poisson regression

*Significant with Bonferroni correction (*p* = 0.00714)

### Correlations with striatal DaT binding

**Fig. 3:**
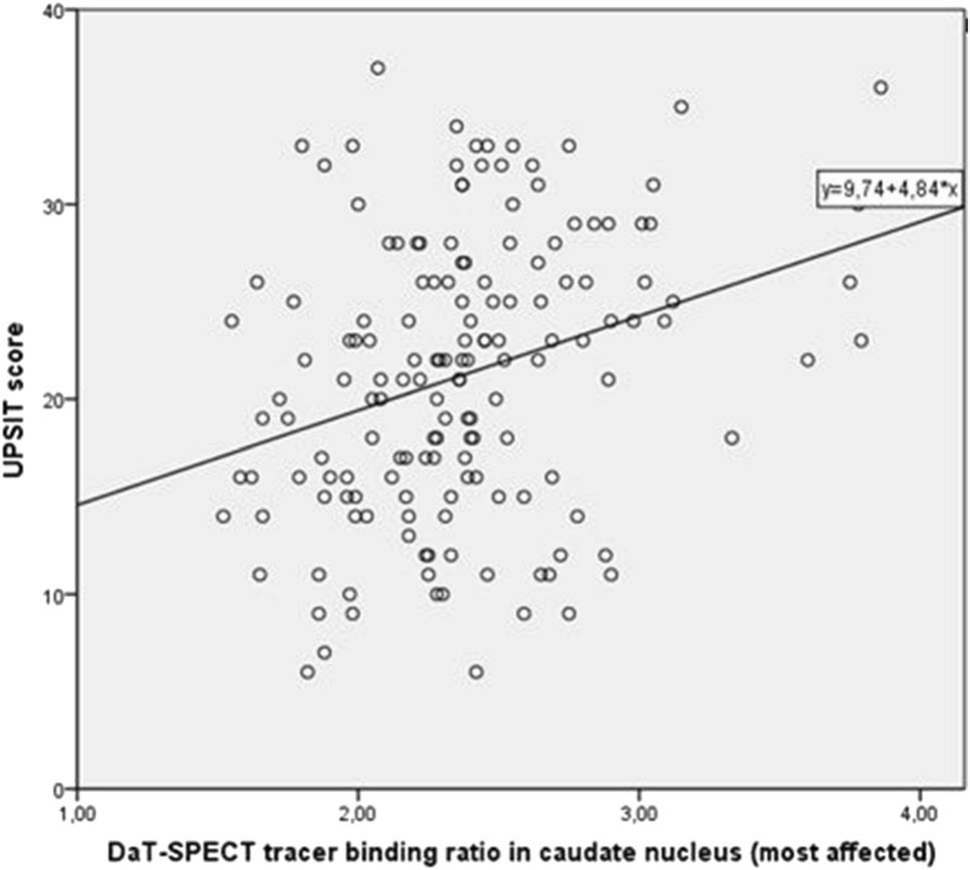
Scatter plot illustrating the correlation between UPSIT scores and striatal DaT-SPECT binding

In the subgroup of patients for whom DaT-SPECT data were available, the prevalence of olfactory dysfunction (i.e., hyposmia or anosmia) was 80%, which was comparable to the prevalence in the total group. The association between UPSIT score and striatal DaT binding in all selected regions was moderate, even after adding sex, age, and disease duration in the analysis, except for the most affected side of the caudate when adding disease duration in the model (Table 3, Fig. 3). Motor function was only weakly associated with striatal binding in all four ROI’s, with the strongest relation between UPDRS III and the least affected side of the putamen. After adjustment for sex, age, and disease duration (models 2 and 3), the association between UPDRS III score and DaT binding was no longer significant in most of the four striatal regions, except in the least affected side of the putamen with the second model. Cognitive function, as measured by the MMSE, had a moderate association with striatal DaT binding in both putamen and caudate nucleus, also in the model adjusted for sex, age, and in all ROI’s except the least affected side of putamen, with disease duration.

**Table 3:** Results of the linear regression analyses relating DaT binding in the putamen to measures of olfactory, motor, and cognitive function

	Most affected side putamen			Least affected side putamen		
	*B*	B (95% CI)	*p*	*B*	B (95% CI)	*p*
UPSIT						
Model 1	0.331	7.634 (4.160; 11.108)	< 0.001*	0.304	5.536 (2.764; 8.309)	< 0.001*
Model 2	0.258	5.957 (2.453; 9.461)	0.001*	0.224	4.086 (1.264; 6.909)	0.005*
Model 3	0.253	5.841 (2.176; 9.516)	0.002*	0.217	3.949 (0.968; 6.930)	0.010*
UPDRS III						
Model 1	− 0.266	− 10.554 (− 16.697; − 4.411)	0.001*	− 0.291	− 9.105 (− 13.925; − 4.286)	< 0.001*
Model 2	− 0.187	− 7.402 (− 13.549; − 1.254)	0.019	− 0.208	− 6.504 (− 11.378; − 1.630)	0.009*
Model 3	− 0.115	− 4.550 (− 10.785; 1.685)	0.151	− 0.131	− 4.115 (− 9.110; 0.880)	0.106
MMSE						
Model 1	0.322	3.337 (1,730; 4.944)	< 0.001*	0.289	2.371 (1.087; 3.655)	< 0.001*
Model 2	0.283	2.932 (1.287; 4.578)	0.001*	0.242	1.980 (0.652; 3.308)	0.004*
Model 3	0.250	2.592 (0.883; 4.301)	0.003*	0.203	1.661 (0.269; 3.052)	0.020

	Most affected side caudate nucleus			Least affected side caudate nucleus		
	*B*	B (95% CI)	*p*	*B*	B (95% CI)	*p*
UPSIT						
Model 1	0.301	4.842 (2.389; 7.295)	< 0.001*	0.329	4.966 (2.687; 7.246)	< 0.001*
Model 2	0.203	3.272 (0.691; 5.852)	0.013*	0.231	3.486 (1.043; 5.930)	0.005*
Model 3	0.192	3.087 (0.395; 5.780)	0.025	0.223	3.363 (0.782; 5.944)	0.011*
UPDRS III						
Model 1	− 0.227	− 6.273 (− 10.601; − 1.946)	0.005*	− 0.256	− 6.659 (− 10.694; − 2.623)	0.001*
Model 2	− 0.115	− 3.171 (− 7.669; 1.327)	0.166	− 0.141	− 3.660 (− 7.930; 0.610)	0.092
Model 3	− 0.036	− 1.002 (− 5.522; 3.519)	0.662	− 0.053	− 1.372 (− 5.725; 2.980)	0.534
MMSE						
Model 1	0.356	2.574 (1.468; 3.679)	< 0.001*	0.352	2.406 (1.359; 3.453)	< 0.001*
Model 2	0.315	2.276 (1.091; 3.461)	< 0.001*	0.308	2.106 (0.969; 3.242)	< 0.001*
Model 3	0.282	2.043 (0.812; 3.274)	0.001*	0.273	1.867 (0.671; 3.063)	0.002*

Model 1: correlation between variable and DaT-SPECT caudate nucleus most affected side (left) and least affected side (right). Model 2: adjusted for age and sex. Model 3: as model 2 plus adjustment with disease duration

*UPSIT* University of Pennsylvania Smell Identification Test, *UPDRS III* Unified Parkinson’s Disease Rating Scale motor subscale, *MMSE* Mini-Mental State Examination

*B *= standardized coefficient. B = unstandardized coefficient

*Significant with Bonferroni correction (*p* = 0.0166)

## Discussion

The present cross-sectional study demonstrates that olfactory function, as measured by the UPSIT, correlates with various motor and non-motor measures of disease severity in PD patients. Especially, the relationship with sleep, depression and anxiety has not been reported previously. This observation emphasizes the profound involvement of the olfactory system in the PD process. In addition, greater olfactory dysfunction was associated with more pronounced loss of nigrostriatal dopamine neurons, as measured by DaT-SPECT, in both the putamen and caudate nucleus. Finally, the data confirm the previously reported high prevalence of olfactory loss in PD patients as measured by the UPSIT (Berendse et al. 2011; Deeb et al. 2010; Doty et al. 1988; Hawkes and Shephard 1998; Hawkes et al. 1997).

It is important to note that the poorest olfactory function was associated with a higher UPDRS III score, corresponding to more severe motor symptoms, even when correcting for sex and age. Only when using a conservative adjustment for multiple testing (Bonferroni), the correlation lost statistical significance. Also previous studies have noted an association between UPDRS III scores and scores on the UPSIT (Berendse et al. 2011; Deeb et al. 2010) and various other olfactory tests (Cavaco et al. 2015; Lee et al. 2015; Masala et al. 2018; Tissingh et al. 2001). The reason why previous studies failed to find an association between olfactory dysfunction and disease severity may reflect differences in study populations, sample sizes, or the types of olfactory tests that were employed (Boesveldt et al. 2008; Doty et al. 1988; Haehner et al. 2009; Herting et al. 2008; Ramjit et al. 2010). A contributory factor is the relative weakness of the association, making it more difficult to detect in studies with insufficient power.

Our finding that disease duration is not independently associated with the degree of olfactory dysfunction is in accord with the findings of earlier studies (Cavaco et al. 2015; Doty et al. 1988; Haehner et al. 2009; Hawkes and Shephard 1998; Herting et al. 2008; Lee et al. 2015; Masala et al. 2018). Thus, olfactory function in PD is largely impacted by disease severity and clinical phenotype, but not by disease duration.

Lower olfactory test scores were correlated in our study with decreased global cognitive function, as measured by the MMSE. This is in line with the results of a previous study in which lower UPSIT scores were associated with more cognitive impairment, as measured by the Montreal Cognitive Assessment (MoCA) test (Fullard et al. 2016). In this study, baseline olfactory test scores were associated with a decline in verbal memory and executive function at follow-up and predicted, in combination with lower Aβ_1–42_ levels, the development of mild cognitive impairment (MCI) within three years. In another study, lower baseline scores on the Brief Smell Identification Test (B-SIT; a test made up of 12 UPSIT items) increased the risk of dementia up to 10 years after PD diagnosis, independent of baseline cognitive performance (Domellof et al. 2017).

The present observation that the severity of the olfactory deficit in PD is associated with the degree of autonomic failure confirms the results of two previous clinical studies in which the olfactory deficit correlated with a higher score on the SCOPA-AUT (Chen et al. 2015; Fullard et al. 2016). In addition, Goldstein et al. reported an association between olfactory test scores and physiological, neurochemical, and neuroimaging markers of autonomic failure in PD (Goldstein et al. 2010). In the present study a higher score on the SCOPA-SLEEP, corresponding to more severe sleeping problems, was associated with a lower UPSIT score. As far as we know, this is the first study to investigate the direct relation between UPSIT scores and sleeping disturbances in PD. However, REM-sleep behaviour disorder has been related to a decline in such scores (Miyamoto et al. 2009; Postuma et al. 2009; Shin et al. 2013; Stiasny-Kolster et al. 2005).

Neuropsychiatric symptoms are highly prevalent in PD (Weintraub and Burn 2011). In a large cross-sectional study by Morley et al., PD patients with lower UPSIT scores exhibited more psychotic symptoms than those with higher UPSIT scores (Morley et al. 2011). Furthermore, measures of apathy reportedly correlate with lower scores on a number of different types of olfactory tests (Cramer et al. 2010; Hong et al. 2015; Masala et al. 2018). However, our study is the first to report a correlation between odor identification test scores and measures of depression and anxiety in PD. A few previous studies have addressed this issue but failed to observe such an association (Rossi et al. 2015; Verbaan et al. 2008), possibly because they employed an olfactory test comprised of only 16 odorants (vs. the 40 of the UPSIT), which may have limited their statistical power.

Our finding of a correlation between olfactory decline and lower striatal DaT binding is in accord with earlier findings (Bohnen et al. 2007; Siderowf et al. 2005). In both studies, this association was demonstrated in PD patients whose disease duration was approximately 2–2.5 years. In our study, the average duration of disease was longer, almost 4 years, implying that the association between smell loss and decreased DaT binding in PD continues over a considerable period of time. Our observation does not necessarily imply that a causal relationship exists between dopaminergic losses and olfactory dysfunction, since numerous other neurotransmitter and neuromodulator systems, including the cholinergic system, are altered in PD (Doty 2017). Moreover, olfactory dysfunction and most other non-motor symptoms do not seem to be influenced by dopaminergic medication (Doty et al. 1988). In addition, despite the largely ipsilateral olfactory afferent projections from the olfactory bulb to the cerebral cortex, there is no association between the side of major motor dysfunction and the side of the nose with greater olfactory dysfunction when, on rare occasion, asymmetry is present (Doty et al. 1992). Lastly, in a PET study of patients with moderately severe PD, cholinergic denervation of the limbic archicortex was more strongly related to poor UPSIT scores than nigrostriatal dopaminergic denervation (Bohnen et al. 2010).

The present study clearly demonstrates that the severity of the olfactory deficit in PD is associated with various motor and non-motor symptoms, as well as with an imaging marker of the integrity of the nigrostriatal dopaminergic system, but not with disease duration. Whether these observations reflect the presence of a group of patients with a more rapid disease progression or suggest that there are PD subtype(s) with more extensive extranigral pathology needs to be further examined in a longitudinal study. Studying olfactory function at baseline and follow-up, in parallel with measuring various motor, non-motor, and imaging markers, may well provide an answer to this longstanding question, as well as confirm the present cross-sectional observations. Olfactory function would have a number of distinct advantages as a clinical marker of disease progression. Not only because of its early-stage presence, but also since olfactory function correlates with both motor and non-motor function and is not influenced by dopaminergic therapy. Moreover, olfactory testing with the UPSIT is non-invasive. Because of its low cost and ability to be self-administered, this test has been found to be very useful in clinical practice.

The strengths of this study include its large sample size, the use of a well-validated and reliable 40-item smell test, and its assessment of the relationship of olfactory test scores of PD patients with a wide range of both motor and non-motor functions, as well as with measures of central dopaminergic function. A potential weakness of the study is that the dosage of dopamine replacement therapy was not taken into account. However, since olfactory function and other non-motor symptoms do not seem to be influenced by dopaminergic medication (Doty et al. 1988), it is unlikely that we have introduced a major bias. Although UPDRS III scores might have been higher in patients examined while not using dopamine replacement therapy, this would likely have increased, not decreased, the observed associations. Another potential weakness is that individuals with other causes of olfactory loss, including trauma or infection, were not specifically excluded from our population. However, any effect would have led to an underestimate of the actual strength of the correlations.

In conclusion, the severity of the olfactory deficit in PD is associated with both clinical (motor and non-motor) and brain imaging measures of disease severity, not with disease duration, per se. Olfactory dysfunction, therefore, merits further longitudinal assessment as a potential clinical marker of disease progression in patients with PD.
